# Systematic characterization of small RNAome during zebrafish early developmental stages

**DOI:** 10.1186/1471-2164-15-117

**Published:** 2014-02-10

**Authors:** Yuangen Yao, Lili Ma, Qiong Jia, Wankun Deng, Zexian Liu, Yuanwei Zhang, Jian Ren, Yu Xue, Haibo Jia, Qing Yang

**Affiliations:** 1 Department of Biomedical Engineering, College of Life Science and Technology, Huazhong University of Science and Technology, Wuhan, Hubei 430074, China; 2 Hefei National Laboratory for Physical Sciences at Microscale and School of Life Sciences, University of Science and Technology of China, Hefei 230027, China; 3 State Key Laboratory of Biocontrol, School of Life Sciences, Sun Yat-sen University, Guangzhou, Guangdong 510275, China

**Keywords:** Deep sequencing, miRNA, piRNA, Zebrafish, Embryonic development

## Abstract

**Background:**

During early vertebrate development, various small non-coding RNAs (sRNAs) such as MicroRNAs (miRNAs) and Piwi-interacting RNAs (piRNAs) are dynamically expressed for orchestrating the maternal-to-zygotic transition (MZT). Systematic analysis of expression profiles of zebrafish small RNAome will be greatly helpful for understanding the sRNA regulation during embryonic development.

**Results:**

We first determined the expression profiles of sRNAs during eight distinct stages of early zebrafish development by sRNA-seq technology. Integrative analyses with a new computational platform of CSZ (characterization of small RNAome for zebrafish) demonstrated an sRNA class transition from piRNAs to miRNAs as development proceeds. We observed that both the abundance and diversity of miRNAs are gradually increased, while the abundance is enhanced more dramatically than the diversity during development. However, although both the abundance and diversity of piRNAs are gradually decreased, the diversity was firstly increased then rapidly decreased. To evaluate the computational accuracy, the expression levels of four known miRNAs were experimentally validated. We also predicted 25 potentially novel miRNAs, whereas two candidates were verified by Northern blots.

**Conclusions:**

Taken together, our analyses revealed the piRNA to miRNA transition as a conserved mechanism in zebrafish, although two different types of sRNAs exhibit distinct expression dynamics in abundance and diversity, respectively. Our study not only generated a better understanding for sRNA regulations in early zebrafish development, but also provided a useful platform for analyzing sRNA-seq data. The CSZ was implemented in Perl and freely downloadable at: http://csz.biocuckoo.org.

## Background

Small non-coding RNAs (sRNAs) of about 20 ~ 30 nucleotides (nt) play an essential role in a variety of animal developmental processes, such as embryonic, neuronal, muscle, and germline development [1-3]. MicroRNAs (miRNAs) and Piwi-interacting RNAs (piRNAs), which are different in biogenesis and biological function, are two predominant types of sRNAs [4,5]. Mature single-strand miRNAs are 21 ~ 25 nt and derived from longer primary miRNA molecules (pri-miRNAs), which are double-stranded RNAs (dsRNAs) and are successively processed by two RNase III endonucleases, namely Drosha and Dicer [1,4]. It was estimated that miRNAs regulate the gene expression of at least one third of all human genes and are involved in a broad spectrum of biological processes, such as development, metabolism, and tumorigenesis by either translational repression, RNA degradation or both through an RNA-induced silencing complex (RISC) [1,2,6,7]. In contrast to miRNAs, piRNAs are slightly longer with a peak size distribution of 26 ~ 28nt, mainly necessary for germ cell maintenance and genome protection by silencing transposable elements [8]. The primary piRNAs are maternally deposited or generated through an unclear mechanism, while more secondary piRNAs are originated from discrete genomic loci termed piRNA clusters, which produce piRNAs from both genomic strands and then reciprocally promote the generation of complementary piRNAs following a Ping-Pong model [9,10].

Analysis of expression profiles of sRNAs is fundamental for understanding the molecular regulations during early vertebrate development [3]. Recent studies in mice revealed an sRNA class transition that the expression levels of piRNAs/siRNAs are gradually reduced while more and more miRNA are expressed during embryonic development [11,12]. This observation was also confirmed in chicken [13] and sea urchins [14]. As a model system, zebrafish has been extensively used as for studying early vertebrate development [15]. Previous studies revealed that most miRNAs are rarely expressed during the first 12 hpf (hours post fertilization) of early zebrafish development but their expression and diversity increase in later stages [16,17]. Although the lack of diversity in early stages, miRNAs still play a crucial role in the regulation of gene expression [2,3,18]. For example, zebrafish miR-430 family is one of the most abundant miRNA families at early developmental stages and leads to degradation and clearance of maternal mRNAs for facilitating the maternal-to-zygotic transition (MZT) [18]. Recently, with the next-generation sequencing (NGS) technique, Wei *et al.* quantitatively analyzed sRNA expression profiles in 256-cell (2.5 hpf), sphere (4 hpf), shield (6 hpf), and 1 dpf (days post fertilization) stages of early zebrafish development [19]. In contrast with previous results, they observed the expressions of both miRNAs and piRNAs are firstly increased and then decreased, with a peak expression at sphere stage for miRNAs and shield stage for piRNAs, respectively [19]. Therefore, more analyses should be carried out to clarify controversial viewpoints of sRNA expression dynamics during zebrafish embryonic development.

Rapid progress in NGS technologies has provided a great opportunity to investigate the sRNA transcriptome at an unprecedented sensitivity [20]. However, it’s still a great challenge to analyze the deep sequencing data in an accurate and fast manner. For sRNA-seq data, characterization of both known and novel miRNAs have attracted most attention because of the functional importance of miRNAs [2]. In contrast with directly mapping reads to known miRNAs for the quantification, prediction of potentially new miRNAs from short reads is particularly difficult and intriguing. In 2005, Xue *et al*. firstly obtained 32 local structure-sequence features (triplet elements) from known human pre-miRNAs and constructed a pre-miRNA predictor of triplet-SVM based on the support vector machines (SVMs) algorithm [21]. Later, MiPred was constructed with a random forest (RF) algorithm by incorporating minimum of free energy (MFE) of the secondary structure of human pre-miRNAs and *P*-value of randomization test with triplet elements in triplet-SVM, with a superior performance than triplet-SVM [22]. Recently, Lertampaiporn *et al*. designed an ensemble predictor of HeteroMirPred by combining a number of machine learning algorithms such as SVMs, RF and *k*-nearest neighbors (kNN), while the prediction results were integrated and selected through a voting system [23]. Previously, by collecting known pre-miRNAs from 133 species, we also developed an SVM-based tool of miRD, which adopted 59 and 139 sequence and structure features for the prediction of single- and multi-stem pre-miRNAs [24]. However, these approaches were designed to predict pre-miRNAs but not mature miRNAs.

Recently, MIREAP (http://sourceforge.net/projects/mireap/files/mireap/) and miRDeep [25] were developed for the identification and prediction of mature miRNAs from the heterogeneous background of high-throughput sequencing data based on the compatibility of miRNA biogenesis model with the distribution characteristics of short reads on candidate precursors. However, the local sequence and structure features of pre-miRNAs were not considered for the prediction. Furthermore, Hackenberg *et al*. constructed an integrative tool of miRanalyzer, which can identify and quantify both known and novel mature miRNAs [26]. To predict novel miRNAs, the RF algorithm and 48 sequence and structure features were used, while known miRNAs for training were taken from human, *C. elegans* and rat. In addition, we also developed a computational platform of CPSS for analyzing the sRNA-seq data, whereas MIREAP and miRDeep were used for the prediction of novel miRNAs [27]. Although a number of efforts have been contributed to this area, no tools were implemented specifically for analyzing zebrafish sRNA-seq data.

In this work, the sRNA-seq technology was first used to determine the expression profiles of sRNAs during eight stages of early zebrafish embryonic development. Based on known zebrafish pre-miRNAs, we designed a zebrafish-specific algorithm of ZmirP (zebrafish miRNA prediction), with 8 new and 57 previously reported sequence and structure features. These features were combined together to construct an SVM model for further filtering potentially false positive hits identified from MIREAP and miRDeep2. The performance and robustness of ZmirP were extensively evaluated by the leave-one-out (LOO) validation and *n*-fold cross-validations. By comparisons on zebrafish-specific pre-miRNAs, ZmirP exhibits greater sensitivity of 95.64% and specificity of 98.84%, which is proved to be better than other existing approaches through comparison. Also, the performance of ZmirP is comparative with other tools for predicting human pre-miRNAs. Then we greatly improved the CPSS [27] and developed a more specific platform as CSZ (characterization of small RNAome for zebrafish) for the analysis of the high-through sequencing data. From the results, we observed that the expression levels of piRNAs are gradually decreased, while miRNA expressions are gradually increased during early embryonic stages. Thus, the sRNA class transition from piRNA to miRNA was confirmed in early zebrafish embryonic development. Furthermore, we observed that the diverse and complex of expression patterns and levels of 129 known miRNA families are dramatically increased as development proceeds. Moreover, 25 novel miRNA candidates were predicted by CSZ with high confidence. We randomly selected three predicted miRNAs for further experimental investigation, and two of them, m0027-5p and chr6_7844-5p, were confirmed through Northern blots. In addition, widespread expression of piRNAs before MZT suggested piRNAs may play a potential role during early development. Taken together, our studies contributed valuable clues for further investigating the sRNA regulation of embryonic development, and provided useful techniques for small RNAome analysis.

## Results and discussion

### A novel algorithm for the prediction of zebrafish-specific pre-miRNAs

To construct a predictive model, we first took 344 known zebrafish pre-miRNAs including 325 single- and 19 multi-stem pre-miRNAs as the positive data set. We also constructed a negative data set containing “pseudo” pre-miRNAs. As previously described [21], the protein coding sequence (CDS) regions were randomly joined together, and fragmented into non-overlapped segments under a constraint condition that the length distribution of extracted segments was identical with that of known zebrafish pre-miRNAs. Then the secondary structures of known zebrafish pre-miRNAs and extracted CDS segments were predicted using RNAfold under the default parameters [28]. To ensure the pseudo pre-miRNAs to be similar with known pre-miRNAs, we randomly selected 325 single- and 19 multi-stem pseudo pre-miRNAs from extracted segments according to two criteria [21]: minimum of 19 base pairings in the hairpins and maximum of −15.79 kcal/mol free energy of secondary structures (including GU wobble pairs). Then based on the training data sets, we used F-score [29] to rank 23 feature sets containing 206 sequence and structure features. Finally, the top 19 sets including 65 features with highest F-score values were selected for constructing the SVMs model (Table 1). The feature set for the prediction of multi-stem pre-miRNAs was not included due to its low F-score.

**Table 1:** Features used in ZmirP

**No.**	**Features**	**Description**
1	%(|G| + |C|)	(|G| + |C|) / L * 100, where L is the length of sequence
2-17	%XY	|XY| / (L − 1) * 100, where |XY| denotes the number of dinucleotide XY, X,Y ⊂ [A,C,G,U]
18	MCPN	Maximum of consecutive paired nucleotides in the secondary structure
19	r_p_n	The total number of paired nucleotides / L
20	r_unp_n	The total number of unpaired nucleotides / L
21	r_p_unp	The total number of paired nucleotides / the total number of unpaired nucleotides
22	avg_bp_stem	The total number of base pairs / the total number of stems^*a*^
23	dP	The total number of base pairs / L
24	n_bulge	The total number of bulges^*b*^
25	r_unp_bulge	The total number of unpaired nucleotides / the total number of bulges
26	r_bulge_l	The total number of bulges / L
27-58	Triplet elements	Triplet structure-sequence elements
59	MFE	Calculated using RNAfold under the default parameters
60	dG	MFE / L
61	MFE1	(MFE / L) /%(|G| + |C|)
62	MFE2	(MFE / L) / n_stems, where n_stems denotes the number of stems
63	MFE3	(MFE / L) / n_loops, where n_loops denotes the number of loops
64	MFE4	MFE / the total number of base pairs
65	MFE5	(MFE / L) / n_bulges

*a*. A stem contains at least three continuous base pairs; *b*. A bulge contains at least three adjacent unpaired nucleotides.

**Figure 1:**
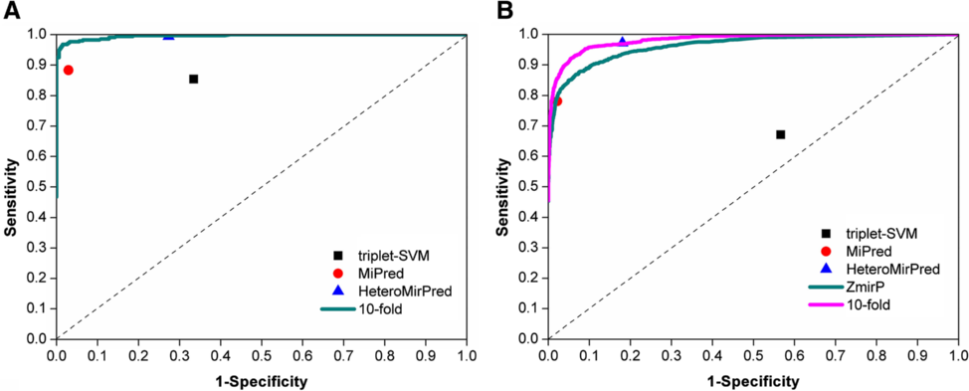
**Comparison of ZmirP with triplet-SVM [**[21]**], MiPred [**[22]**] and HeteroMirPred [**[23]**].** To evaluate the performance ZmirP, the 10-fold cross-validations were performed. For the comparison, we directly submitted the training data set to other tools for calculating the performance values. **(A)** ZmirP with zebrafish-specific; **(B)** ZmirP with human-specific.

To evaluate the performance of the ZmirP algorithm, the LOO validation and 4-, 6-, 8-, 10-fold cross-validations were performed. Because the results of LOO validation and *n*-fold cross-validations are were similar, only the ROC curve of 10-fold cross-validation was visualized (Figure 1A). Furthermore, we compared ZmirP to several other existing tools, including triplet-SVM [21], MiPred [22], and HeteroMirPred [23]. The training dataset used in ZmirP was directly submitted into other tools for the prediction. Then we fixed the *Sp* values of triplet-SVM, MiPred and HeteroMirPred to be identical with ZmirP and compared the *Sn* values. When the *Sp* value was 66.57%, the *Sn* values of ZmirP and triplet-SVM were 99.71% and 85.47%, respectively (Table 2). When the *Sp* value was 97.09%, the *Sn* values of ZmirP and MiPred were 95.93% and 88.37%, respectively (Table 2). In addition, when the *Sp* value was 72.67%, the *Sn* values of ZmirP and HeteroMirPred were 99.71% and 99.42%, respectively (Table 2). Thus, the comparison results suggested that ZmirP is more accurate than other predictors for zebrafish pre-miRNAs (Figure 1A). To avoid any bias, we also compared ZmirP to other approaches by using 1,600 human pre-miRNAs as a positive data set. A negative data set containing 1,600 “pseudo” pre-miRNAs were constructed from human CDS regions. We directly inputted this independent data set into ZmirP and other tools, whereas the results suggested that the performance of ZmirP is better than triplet-SVM and comparative with MiPred and HeteroMirPred (Figure 1B). Because ZmirP was trained with Zebrafish-specific pre-miRNAs and the other three tools used human pre-miRNAs for training, we re-trained the SVM model of ZmirP with human pre-miRNAs. The 10-fold cross-validation result exhibited that our approach is better than triplet-SVM and MiPred, but the accuracy is slightly lower than HeteroMirPred (Figure 1B).

**Table 2:** Comparison of ZmirP with other approaches for zebrafish pre-miRNAs

**Method**	***Ac *** **(%)**	***Sn *** **(%)**	***Sp *** **(%)**	***MCC***
**ZmirP**	97.24	95.64	98.84	0.9453
	83.14	99.71	66.57	0.7025
	96.51	95.93	97.09	0.9303
	86.19	99.71	72.67	0.7518
**triplet-SVM**	76.02	85.47	66.57	0.5299
**MiPred**	92.73	88.37	97.09	0.8579
**HeteroMirPred**	86.05	99.42	72.67	0.7482

The dataset used in ZmirP was directly submitted in other tools. Then we fixed the *Sp* values of ZmirP to other predictors and compared the *Sn* values.

### Development of the CSZ platform for analyzing zebrafish sRNA-seq data

**Figure 2:**
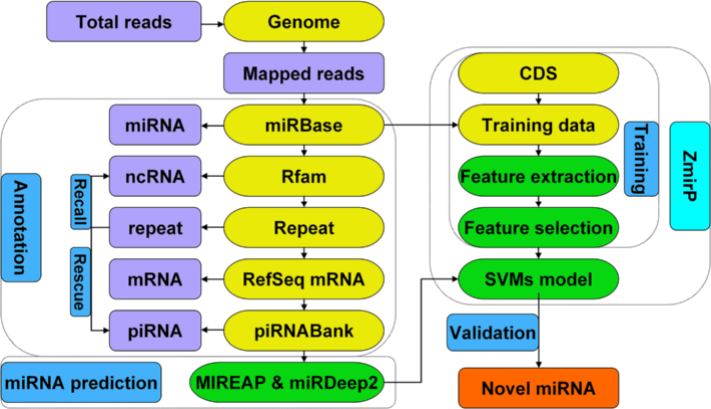
**The computational pipeline in CSZ.** First, total reads were mapped to reference genome, while mapped reads were successively mapped to miRBase, Rfam, repeat annotations, RefSeq mRNAs, and piRNABank to identify miRNAs, ncRNAs (including rRNA, tRNA, and snRNA/snoRNA), repeats, mRNAs and piRNAs. Based on the annotation information for genomic repeats, the ncRNAs were recalled and repeat-associated piRNAs were characterized from remaining repetitive sequences. For the unclassified reads, MIREAP and miRDeep2 were used for the prediction of novel miRNAs, which were further validated by ZmirP to reduce potentially false positive hits.

In this work, we greatly improved CPSS and developed a more efficient platform as CSZ for characterizing small RNAome from the deep sequencing data in zebrafish [27] (Figure 2). First, we only reserved unique reads ranging from 18 to 35 nt. The short reads observed with at least three times were assumed to be potential sRNA molecules or degradation fragments of larger RNAs [30], and were mapped to the zebrafish reference genome using Bowtie, with only one nucleotide mismatch [31]. Then the identified reads were subsequently mapped to miRBase, Rfam, repeat sequences, RefSeq mRNAs, and piRNABank (Figure 2). By this procedure, the reads were successively classified into the following categories, including miRNA, rRNA, tRNA, and snRNA/snoRNA, genomic repeat, mRNA, and piRNA. Because repeat sequences in the annotation file were pre-classified into different classes, such as rRNA, tRNA, and snRNA/snoRNA, these RNAs were removed from the repeat sequences and recalled back to the four groups (Figure 2). Also, because piRNAs can locate in repetitive sequences, we further identified potential piRNAs by mapping other repetitive sequences to piRNABank (Figure 2). As previously described [27], the observed frequency of a multi-mapped reads was divided by the number of its mapping positions. To identify the expression profiles of sRNAs among different samples, the observed frequencies of unique sequences were normalized to the reads per million (RPM) data [27].

In our results, a large proportion of reads were identically mapped to known miRNAs in miRBase, but with a few number of shorter or longer nucleotides. These sequences might be produced by imprecise Dicer processing and non-template-directed nucleotide addition, and stand for different isoforms of the same miRNA, which were referred as isomiRs [30]. Therefore, a small window around the location of an annotated pre-miRNA plus 2 nt upstream and 5 nt downstream, was tolerated through mapping unique reads to known pre-miRNAs [32]. Although different isomiRs existed for one miRNA, the observed frequencies of most abundant isomiRs were adopted as the expression levels of sequenced miRNAs [30]. For unknown sequences that could not be assigned to any of known categories, we detected potentially novel miRNAs using MIREAP (https://sourceforge.net/projects/mireap/) and miRDeep2 [32] with the default settings. Because too many putative results were generated by MIREAP or miRDeep2, we adopted the ZmirP algorithm for further filtering potentially false positive hits, with a default cut-off value of 0.8.

### Systematic analysis of small RNAome in early zebrafish development

**Figure 3:**
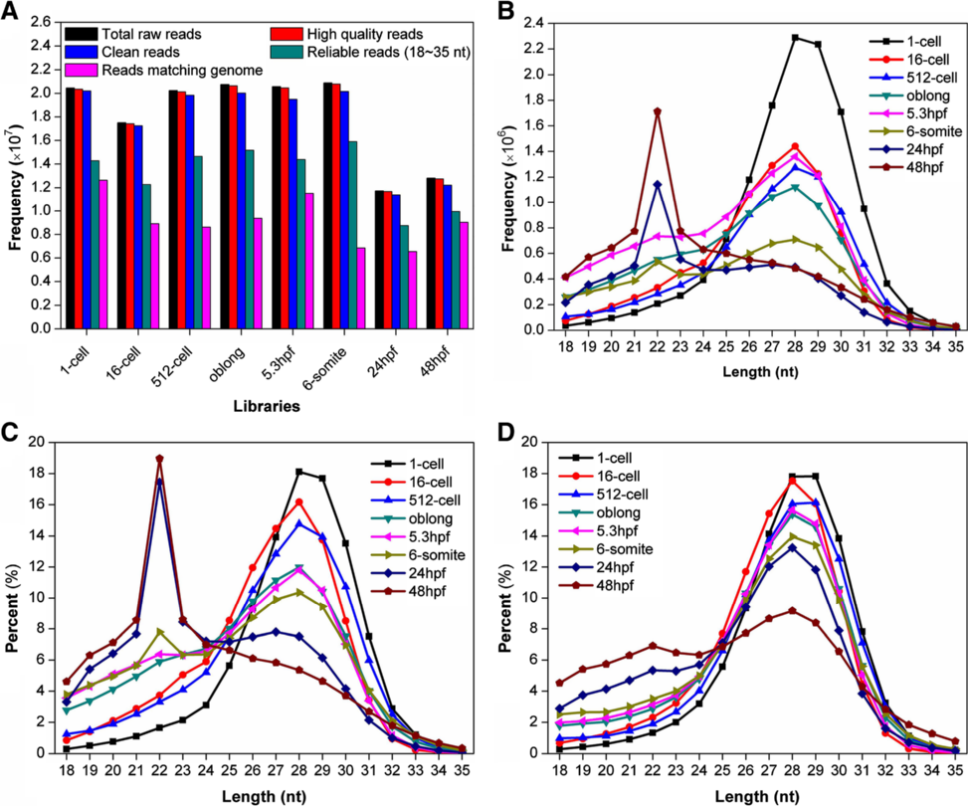
**The summary of sRNA-seq data. (A)** Number of different types of reads in eight libraries. High quality reads represent reads without N characters, or without quality scores lower than 10 for > 4 bases or 13 for > 6 bases. Clean reads represent reads without adaptors and contaminants. Reads with size ranging from 18 to 35 nt and observed more than three times was considered as reliable reads. Only one nucleotide mismatch was allowed for mapping reads to the reference genome; **(B)** The length distribution of mappable reads in eight libraries; **(C)** The proportion of total mappable reads with different length during development; **(D)** The proportion of unique reads with different length.

The total RNAs during eight distinct stages, including 1-cell (0.2 hpf), 16-cell (1.5 hpf), 512-cell (2.75 hpf), oblong (3.7 hpf), 5.3 hpf, 6-somite (12 hpf), 24 hpf and 48 hpf, of zebrafish early embryonic development were isolated and used for the sRNA-seq. To identify the expression profiles of sRNAs, the sRNA-seq was implemented in the Illumina platform, which produced 20,450,552, 17,504,132, 20,235,715, 20,753,650, 20,568,988, 20,893,594, 11,739,974 and 12,823,319 raw reads in eight libraries, respectively (Figure 3A, Additional file 1: Table S1). After removal of low quality reads, the eight libraries included 20,346,737, 17,411,182, 20,126,917, 20,634,984, 20,456,205, 20,778,822, 11,681,893 and 12,752,467 high quality reads, respectively (Figure 3A, Additional file 1: Table S1). We also cleared adaptor sequences, contaminated sequences and sequences containing poly(A) tails to obtain clean reads (18 ~35 nt) observed with at least three times and unique tags (Figure 3A, Additional file 1: Table S1). Finally, the reads matching the reference genome with one mismatch tolerance were 12,640,308, 8,911,507, 8,617,713, 9,370,664, 11,520,438, 6,848,228, 6,552,034 and 9,031,347, and accounted for 61.81%, 50.91%, 42.59%, 45.15%, 56.01%, 32.78%, 55.81%, 70.43% of total raw reads in corresponding libraries, respectively (Figure 3A, Additional file 1: Table S1).

The analysis of the length distribution of reads matching the reference genome within each developmental stage uncovered two peaks between 18 nt and 35 nt (Figure 3B). The first peak around 22 nt is a potentially strong signal for miRNAs according to their characteristic of size distribution, while the second broader peak around 28 nt may represent piRNAs (Figure 3B). Because different samples had different sequencing depths, we took the factor into account by normalizing observed frequencies of reads into RPM data, while the relative abundances of reads with different lengths in eight developmental stages were illustrated under the same scale. Again, the bimodal length distribution with two different peaks around 22 nt and 28 nt was robustly detected (Figure 3C). Interestingly, we also observed that the percentiles of ~22-nt sequences dramatically increased from ~1.64% of 1-cell to ~18.96% of 48hpf (~11.5 fold), while the proportions of ~28-nt sequences significantly decreased from ~18.11% in 1-cell to ~5.36% in 48hpf (~3.4 fold) (Figure 3C). In addition, to analyze the diversity of sRNA sequences, the relative abundances of unique sequences with different lengths also were visualized (Figure 3D). Although the peak around 22 nt was not very significant, the percentiles still increased from ~1.32% of 1-cell to ~6.91% in 48hpf (~5.2 fold) (Figure 3D). Thus, our results demonstrated that both miRNA abundance and diversity dramatically increased during the early development. In contrast, the second peak around 28 nt was not influenced, and the results suggested that both abundance and diversity of piRNAs significantly decreased from 1-cell stage to 48 hpf (Figure 3D). Taken together, our analyses clearly demonstrated an sRNA transition from piRNA to miRNA during zebrafish early embryonic development.

### Expression profiles of known and novel miRNAs in zebrafish early embryonic development

**Figure 4:**
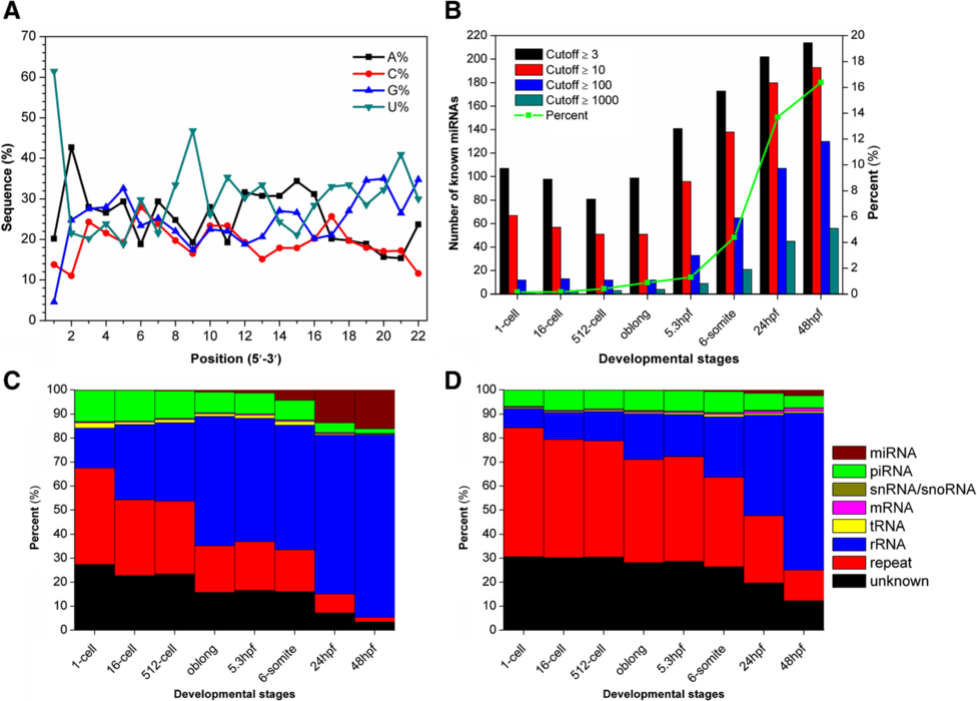
**The analyses of known miRNAs in early zebrafish development. (A)** The nucleotide preferences of 218 identified miRNAs; **(B)** The identified number of miRNAs under different thresholds for mapped reads (≥ 3, ≥ 10, ≥ 100, and ≥ 1000). The proportion of miRNAs in total sRNA-seq data was also shown; **(C)** The proportion of total mappable reads for different types of sRNAs; **(D)** The distribution of unique mapped reads for different types of sRNAs.

From the sRNA-seq data, we totally detected 218 mature miRNAs of total 247 known zebrafish miRNAs in miRBase, with at least three mapped reads (Additional file 2: Table S2). Previous studies reported that the first and ninth nucleotides at the 5′ end of metazoan miRNAs are frequently uracil (U) [33]. To evaluate this viewpoint in zebrafish, the nucleotide distribution of 218 identified miRNAs was analyzed at each position (Figure 4A). As expected, there was a high frequency of U nucleotides at positions one (61.47%) and nine (46.79%), whereas the guanine (G) nucleotide was rarely observed at position one (4.59%) (Figure 4A). Moreover, an excess of U + G nucleotides occurred from position 17 to the 3′ end of sequences (Figure 4A). Generally, position-specific preferences in zebrafish miRNA sequences were consistent with that in other metazoan miRNA sequences [33].

By using different thresholds for mapped reads (≥ 3, ≥ 10, ≥ 100, and ≥ 1000), we observed that less miRNAs were observed before 5.3 hpf and the number of miRNAs rapidly increased after the stage (Figure 4B). Using a cutoff value of ≥ 3, the numbers of known miRNAs in eight samples were 107, 98, 81, 99, 141, 173, 202 and 214, respectively. When the threshold was increased to 100, the number of known miRNAs in eight samples significantly dropped to 12, 13, 12, 12, 33, 65, 107 and 130, respectively. Thus, the results demonstrated most miRNAs are lowly expressed before 5.3 hpf (Figure 4B). Moreover, the percentages of miRNAs in sRNA-seq data were also shown, while the expression curve further confirmed that the miRNA abundance increased more significantly than the miRNA diversity, especially after the 5.3 hpf stage (Figure 4B). In addition, the distribution of different types of sRNAs was shown for total reads (Figure 4C) and unique reads (Figure 4D). The results not only confirmed the piRNA to miRNA transition, but also demonstrated that miRNA abundance enhanced more dramatically than the diversity. Take together, the expression levels of miRNAs were largely determined by a limited number of potentially important miRNAs.

**Figure 5:**
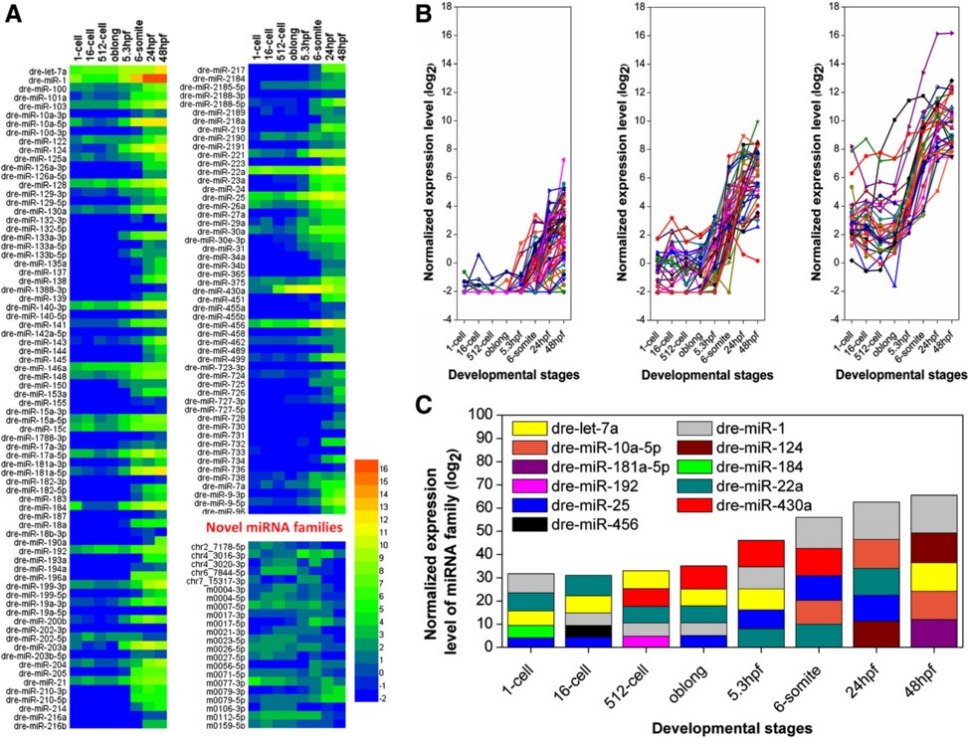
**The clustering analysis of miRNA families. (A)** The RPM-normalized expression profiles of known and novel miRNA families in eight developmental stages; **(B)** The RPM-normalized expression profiles of known miRNA families were clustered into three distinct groups with the *k*-means clustering algorithm in Cluster 3.0 [35]; **(C)** Top 5 mostly expressed miRNA families were shown for each stage.

Based on the conservation of seed sequences (2–8 nt), we classified 218 known miRNAs into 129 miRNA families, while the RPM-normalized expression profiles were visualized for eight distinct developmental stages (Figure 5A). Also, integrated analyses of all sRNA-seq data during eight developmental stages with the CSZ platform identified 25 novel miRNAs that had not been previously reported (Table 3, Additional file 3: Table S3). These novel miRNAs were classified into 22 families and their expression profiles were shown (Figure 5A). Before the 5.3 hpf, most known miRNA families were lowly expressed and the expression diversity was not high. However, both the expression levels and diversity of known miRNA families were rapidly increased after the stage (Figure 5A). Thus, although the MZT starts from the 512-cell stage in zebrafish [15], the significant changes of known miRNAs are delayed because the simultaneous degradation of maternal miRNAs and synthesis of zygotic miRNAs [34]. For novel miRNA families, such a dramatic change around the 5.3 hpf was not observed. Most of the novel miRNAs are highly expressed in specific stages, and such an analysis is consistent with previous studies [19]. Also, the expression profiles of known miRNA families were clustered into three distinct groups with the Cluster 3.0 [35]. The first group included 61 miRNA families that are expressed at low levels across all eight developmental stages (Figure 5B). The second group was composed of 38 miRNA families that are lowly expressed before the 5.3 hpf, while the expressions are dramatically increased in later stages (Figure 5B). However, the last group contained 30 miRNA families that were expressed at higher levels before the MZT onset (512-cell) [15], followed by a remarkable increase at the oblong stage (Figure 5B). Furthermore, the top 5 most abundant miRNA families expressed at each stage were selected, including dre-let-7a, dre-miR-1, dre-miR-10a-5p, dre-miR-124, dre-miR-181a-5p, dre-miR-184, dre-miR-192, dre-miR-22a, dre-miR-25, dre-miR-430a and dre-miR-456 families (Figure 5C). All these mostly expressed miRNAs were included in the last group of miRNA families. Thus, the miRNAs in the last group might be maternally inherited and play a potential role in the MZT.

**Table 3:** Totally, we predicted 25 potentially novel miRNAs with high confidence

**Name**	**1-cell**	**16-cell**	**512-cell**	**oblong**	**5.3hpf**	**6-somite**	**24hpf**	**48hpf**	**ZmirP**^*a*^
m0086-5p	0	0	0	2.0	16.0	37.7	22.0	9.5	1.0000
m0017-3p	20.0	14.0	15.0	11.0	31.0	21.0	18.0	3.0	1.0000
m0017-5p	0	0	0	1.0	7.0	37.7	22.0	9.3	1.0000
m0059-5p	0	3.0	3.0	3.0	16.0	37.7	22.0	10.0	1.0000
m0056-5p	0	0	0	0	4.0	49.0	25.0	26.0	1.0000
m0021-3p	4.0	8.0	30.0	32.0	37.0	19.0	0	0	0.9997
chr2_7178-5p	27.0	36.0	22.0	17.0	6.0	10.0	3.0	0	0.9979
m0079-3p	6.0	4.0	5.0	4.0	10.0	19.0	84.0	191.0	0.9971
m0079-5p	14.0	31.0	31.0	26.0	43.0	45.0	121.0	228.0	0.9971
m0004-5p	20.0	41.0	44.0	30.0	19.0	16.0	6.0	5.0	0.9957
m0077-3p	469.0	122.0	96.0	66.0	39.0	20.0	62.0	32.0	0.9904
m0026-5p	63.0	47.0	37.0	22.0	46.0	37.0	9.0	9.0	0.9849
m0024-5p	35.0	30.0	26.0	26.0	13.0	5.0	9.0	0	0.9759
m0071-5p	45.0	29.0	30.0	24.0	14.0	34.0	8.0	6.0	0.9741
m0106-3p	4.0	33.0	32.0	28.0	4.0	0	6.0	0	0.9669
chr6_7844-5p	7.0	130.0	116.0	82.0	39.0	4.0	4.0	0	0.9629
m0159-5p	39.0	52.0	26.0	23.0	21.0	7.0	7.0	0	0.9528
m0027-5p	25.0	41.0	19.0	28.0	4.0	10.0	0	0	0.9526
m0007-5p	108.0	47.0	72.0	65.0	82.0	67.0	17.0	11.0	0.8997
chr4_3020-3p	3.0	23.0	36.0	37.0	6.0	85.0	6.0	4.0	0.8695
m0023-5p	4.0	18.0	20.0	24.0	5.0	5.0	0	0	0.8578
chr4_3016-3p	68.0	12.0	18.0	32.0	445.0	98.0	137.0	28.0	0.8504
m0004-3p	16.0	17.0	53.0	44.0	22.0	14.0	7.0	3.0	0.8273
m0112-5p	81.0	114.0	93.0	78.0	104.0	57.0	12.0	6.0	0.8247
chr7_15317-3p	34.0	4.0	5.0	3.0	67.0	33.0	3.0	0	0.8015

The number of reads at each developmental stage was provided. *a*. The prediction score of ZmirP algorithm.

### Experimental validation of known and novel miRNAs

**Figure 6:**
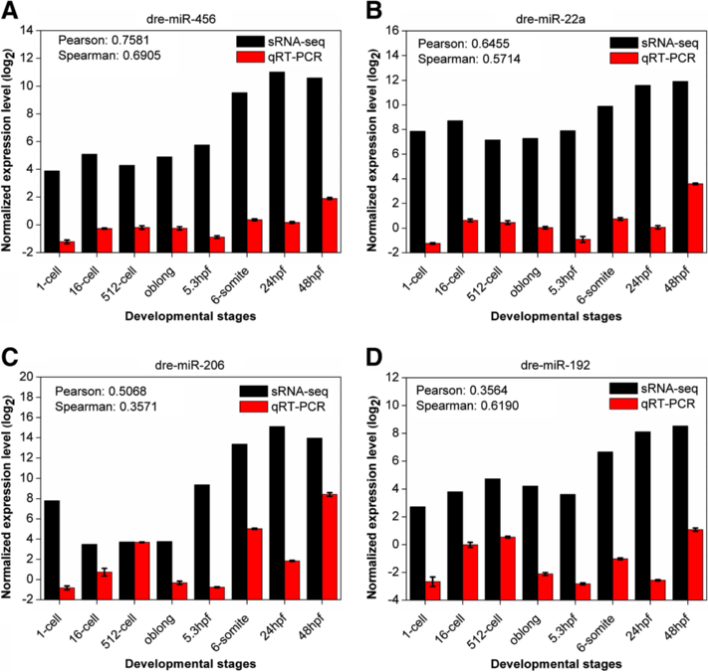
**Experimental validation of expression profiles for four know miRNAs with qRT-PCR.** Each experimental validation was repeated three times, whereas the error bars were added for qRT-PCR experiments. **(A)** dre-miR-456; **(B)** dre-miR-22a; **(C)** dre-miR-206 and **(D)** dre-miR-192.

To verify the expression profiles of known miRNAs, we selected four miRNAs (dre-miR-456, dre-miR-22a, dre-miR-206, and dre-miR-192) from the last group based on their known or potential roles in zebrafish early development. Previously, the experiments in chicken identified that the miR-456 is essential for maintaining the undifferentiated state of the blastoderm before MZT [36], and we observed the dre-miR-456 family was highly expressed in the 16-cell stage (3.66%) (Figure 5C). Mouse miR-22 was reported to regulate cell cycle progression in cerebellum development [37]. In our results, the dre-miR-22a family was highly expressed through the first seven stages from 1-cell (27.41%) to 24hpf (2.93%) (Figure 5C). The dre-miR-206 is a member of zebrafish miR-1 family, which control angiogenesis by regulating VegfA expression during embryonic development [38] and abundantly expressed throughout all eight stages (Figure 5C). In addition, the dre-miR-192 family was reported to be involved in zebrafish immune response [39], where our results observed the family is over-expressed in 512-cell (3.27%) (Figure 5C). The expression levels of the four miRNAs were validated by qRT-PCR at eight developmental stages (Figure 6). Previously, the comparison of Illumina sequencing and qRT-PCR only generated a moderate correlation, mainly due to the sequencing bias or post-transcriptional modifications of miRNAs [40]. In our results, the four miRNAs exhibited promising Pearson or Spearman correlation coefficients between sRNA-seq and qRT-PCR data (Figure 6A, B, C, & D). Thus, our experiments confirmed that identified known miRNAs are differentially expressed during early developmental stages.

**Figure 7:**
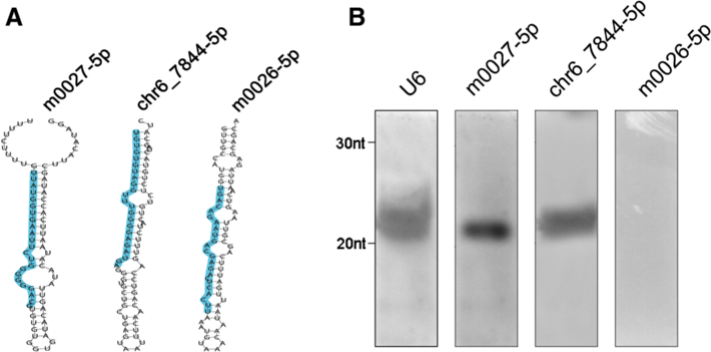
**Confirmation of potentially novel miRNAs through non-isotopic northern blots. (A)** The analyses of secondary structures for m0027-5p, chr6_7844-5p and m0026-5p revealed that the three sRNAs had canonical single stem-loop structures. The potentially mature miRNAs were marked in blue. **(B)** The experiments identified that m0027-5p and chr6_7844-5p are expressed in zebrafish 16-cell stage.

Besides known miRNAs, we also predicted 25 potentially novel miRNAs in zebrafish (Table 3, Additional file 3: Table S3). Using RNAfold with the default parameters, we observed that all these miRNAs have canonical single stem-loop structures (Additional file 4: Figure S1). From the predictions, we randomly selected three hits, including m0027-5p, chr6_7844-5p and m0026-5p for further experimental validations (Figure 7A). The non-isotopic Northern blotting analyses demonstrated that m0027-5p and chr6_7844-5p are expressed in zebrafish 16-cell stage (Figure 7B). Take together, our experimental verifications on both known and novel miRNAs indicated that CSZ is an accurate and efficient platform.

### Expression levels of piRNAs in zebrafish early development

**Figure 8:**
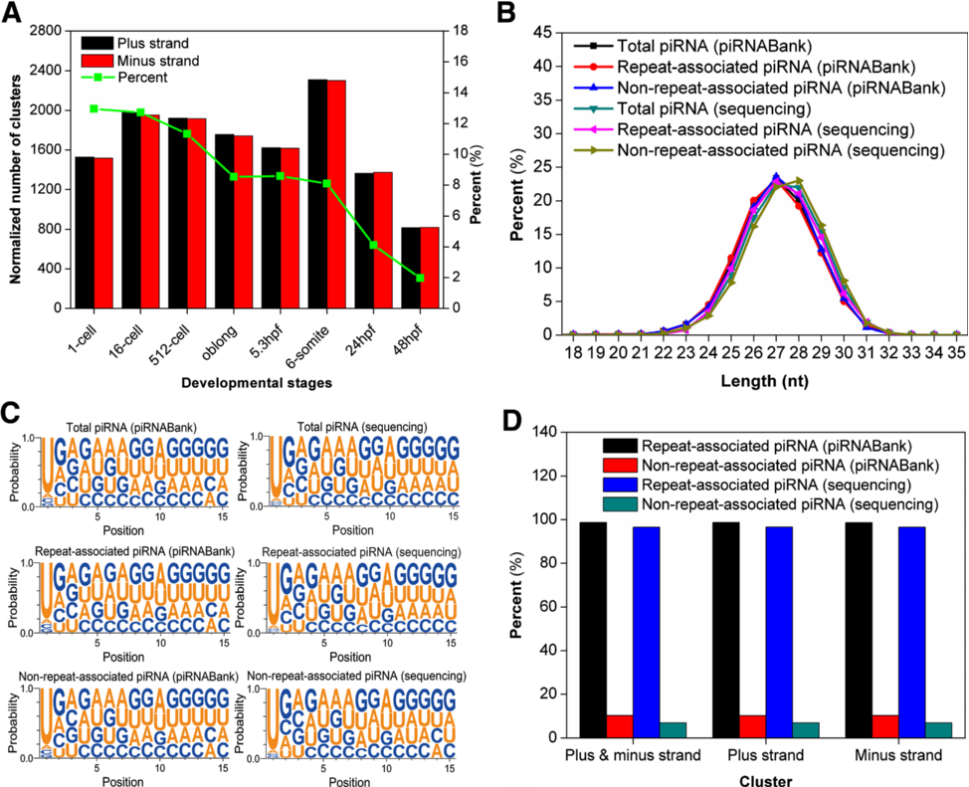
**Computational analysis of piRNAs. (A)** The normalized number of piRNA clusters in either plus or minus strand was calculated for each stage; **(B)** The length distribution of piRNAs from different sources; **(C)** The sequence logos of piRNAs from different sources; **(D)** The distribution of piRNA clusters originated from repeat-associated or non-repeat-associated piRNAs.

The majority of piRNAs locate in genome as clusters of length 20-100 kb, and it was suggested that piRNAs are originated from long transcripts then subsequently processed into ~28 nt smaller RNAs [9,41]. As previously described, the piRNA clusters were defined as genomic regions containing at least 10 unique piRNA loci and the distance between two adjacent piRNA loci with less than 1 kb [19] (Additional file 5: Table S4). By clustering identified piRNAs into clusters, the expression abundance and diversity of piRNAs in each developmental stage were analyzed (Figure 8A). Since most piRNAs are maternally deposited and gradually degraded as development proceeds [13], we observed a reduction of piRNA expression throughout the eight stages (Figure 8A). However, piRNAs are essential for inhibiting the activities of transposable elements, and a considerable proportion of piRNAs are zygotically expressed rather than maternally inherited [12,13]. As expected, we found the diversity of piRNAs was first increased then dramatically decreased (Figure 8A). In addition, by analyzing the genomic locations of identified piRNAs, we revealed that piRNAs were equally located at either plus or minus strands (Figure 8A).

Previously, we only considered piRNAs that were not annotated as repeat sequences (Non-repeat-associated piRNAs) in CPSS [27]. However, over 50% of piRNAs in both piRNABank database and our results could be mapped to repetitive sequences (Repeat-associated piRNAs). Also, by analyzing the length distribution, we observed that piRNAs from different sources were very similar (Figure 8B). Furthermore, the nucleotide preferences of 15-nt subsequences taken from 5′-end sequences of piRNAs were visualized by WebLogo 3 [42]. No significant differences were observed between repeat-associated and non-repeat-associated piRNAs (Figure 8C). In addition, we separately grouped repeat-associated and non-repeat-associated piRNAs from piRNABank and our results into distinct clusters, and revealed that ~90% piRNA clusters were derived from repeat-associated piRNAs (Figure 8D). In this regard, the rescue of piRNAs from unclassified repeat sequences is essential for a more comprehensive analysis of piRNAs. Taken together, although piRNAs were proposed to be functional and abundantly produced in germline cells, highly expressed piRNAs before the MZT suggested that a considerable number of piRNAs might play an important role in zebrafish early embryonic development.

## Conclusions

During early vertebrate development, various sRNAs are temporally and spatially expressed to orchestrate the embryogenesis [3]. Previous studies in several model organisms revealed an sRNA class transition from piRNAs/siRNAs to miRNAs, and the transition is essential for the MZT by activating zygotic genome and clearing maternal RNAs [11-14,18]. However, this phenomenon was not observed from a recent study of four developmental stages in zebrafish [19]. To evaluate the viewpoint, here we systematically characterized the transcriptional profiles of zebrafish sRNAs with the sRNA-seq technology from eight early developmental stages, including 1-cell, 16-cell, 512-cell, oblong, 5.3 hpf, 6-somite, 24 hpf and 48 hpf. To promise the quality for mapping reads, only one nucleotide mismatch was permitted for all sequence alignments. After removing low quality reads and uninformative sequences, we totally mapped 32.78% ~ 70.43% of all reads to the zebrafish genome (Additional file 1: Table S1).

How to retrieve useful information from the huge amount of repeat sequences is a great challenge for analyzing the sRNA-seq data. Many types of RNAs were annotated in repetitive sequences, such as rRNA, tRNA, snRNA/snoRNA, and repeat-associated piRNAs. If these annotations were not considered, the identification of the five types of sRNAs would be greatly underestimated. Thus, we greatly optimized our previous pipeline and designed a more efficient platform of CSZ, which first recalled rRNA, tRNA, and snRNA/snoRNA back to their own groups and then re-characterized piRNAs from the remaining repeats. For predicting potentially novel miRNAs, we also greatly refined our algorithm. Totally, there were 197 sequence and structure features used in miRD for the prediction of single- or multi-stem pre-miRNAs [24]. However, only 65 features were used in ZmirP, and only two features including MFE and the ratio of paired nucleotide to unpaired nucleotide were share by the two methods. Furthermore, because only small proportion of multi-stem pre-miRNAs exists in zebrafish, we used all known zebrafish pre-miRNAs for training. Indeed, we did not observe any multi-stem pre-miRNAs in 25 newly predicted results (Table 3, Additional file 4: Figure S1). By comparison, ZmirP exhibited a superior performance for predicting zebrafish pre-miRNAs (Figure 1A), and can be comparable with than other existing tools for human pre-miRNAs (Figure 1B). Because several steps in CSZ, such as the identification of potential miRNAs by MIREAP/miRDeep2 and reads mapping to genome, were too time-consuming, the development of a web server will be a heavy burden for our computational resources. Thus, the CSZ was written in Perl as a stand-alone package at: http://csz.biocuckoo.org/down.php. The SVM model for zebrafish pre-miRNAs was directly included in the program, while the human-specific model was also provided. Take together, the newly developed platform can accurately identify both miRNAs and piRNAs from the sRNA-seq data.

**Figure 9:**
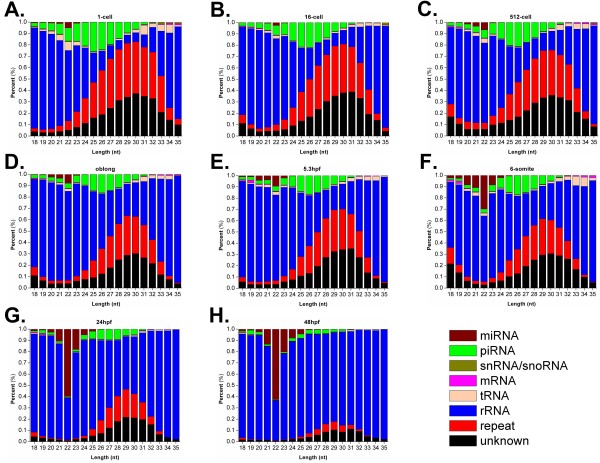
**The length distribution of different types of sRNAs at eight stages. (A)** 1-cell, **(B)** 16-cell, **(C)** 512-cell, **(D)** oblong, **(E)** 5.3 hpf, **(F)** 6-somite, **(G)** 24 hpf and **(H)** 48 hpf stages.

From the results, we clearly observed a piRNA to miRNA transition during early zebrafish embryonic development (Figure 3B, 3C, 3D, Figure 4C, and D). Additionally, the length distribution of different types of sRNAs at each stage was analyzed (Figure 9). The results further confirmed that an increase of miRNA expression and a reduction of piRNA levels as early development proceeds (Figure 9). However, miRNAs and piRNAs had distinct dynamics on abundance and diversity. Both miRNA expression levels and diversity dramatically increased during development, while its abundance was enhanced more significantly than the diversity. However, although the expression of piRNAs is gradually decreased, their diversity first increases then rapidly decreased because a small proportion of zygotic piRNAs are also expressed. Therefore, our analysis in zebrafish confirmed the sRNA class transition is a conserved mechanism in metazoans [11-14,18]. We also re-analyzed the dataset released by Wei *et al.*[19] from the GEO database with the accession number of GSE27722. However, the piRNA-miRNA transition was not observed for either sRNA abundance (Additional file 6: Figure S2A) or diversity (Additional file 6: Figure S2B). In Wei’s results [19], the miRNA abundance can occupy up to ~83% of total sRNAs at sphere stage (Additional file 6: Figure S2A), whereas the miRNA abundance of our results only reached ~16% at 48hpf (Figure 4C). Previously studies in mouse [12] and sea urchins [14] suggested that miRNAs are lowly expressed in early stages and the expression levels gradually increase as development proceeds. And the piRNA-miRNA transition has been confirmed in multiple species [11-14]. Since even our approach can not detect such a transition from Wei’s data, we proposed that the quality of their RNA library might not be high enough and still need to be optimized.

Our analyses on known miRNAs expressed in early development are consistent with previous studies in zebrafish or other model organisms. For example, as one of the most abundant miRNA families, zebrafish miR-430 family facilitates the MZT by the clearance of maternal mRNAs [18]. In our results, the dre-miR-430a family (including dre-miR-430a, dre-miR-430b, dre-miR-430c and dre-miR-430i) is highly expressed from 512-cell to 6-somite stage, and may be functional in MZT (Figure 5C). Also, our results of dre-miR-456 and dre-miR-22a families are also consistent with the studies in the early development of chicken [36] and mouse [37], respectively. Moreover, we identified a number of known miRNAs reported to be involved in other processes may also play a potential role in embryonic development. For example, the dre-miR-192 family was characterized to be implicated in zebrafish immune response to bacterial infection [39], whereas our results suggested that the family may also participate in regulating the early development (Figure 5C). In addition, we predicted 25 potential novel miRNAs, and validated two of them, m0027-5p and chr6_7844-5p, are expressed in zebrafish 16-cell stage samples (Figure 7). Taken together, our results can be a useful resource for further analysis of sRNAs in early zebrafish embryonic development.

## Methods

### Data preparation

The zebrafish genome assembly version 2010 (Zv9) was downloaded from the UCSC database [43]. Sequences of 247 zebrafish mature miRNAs and 344 pre-miRNAs were obtained from miRBase (release 19) [44]. Also, zebrafish snRNA, snoRNA, tRNA and rRNA were retrieved from Rfam (release 11.0) [45]. Zebrafish repeat sequences were extracted from the reference genome according to the annotations in the UCSC database [43]. Moreover, the RefSeq mRNAs of zebrafish were downloaded from the UCSC database [43]. The zebrafish piRNAs were retrieved from the piRNABank, a comprehensive data resource for piRNAs [41]. And the protein coding sequences (CDSs) of zebrafish were obtained through the Table Browser in the UCSC database [43]. In addition, we obtained 1,600 human pre-miRNAs from miRBase (release 19) [44].

### The ZmirP algorithm

Previously, we selected different features to predict single- and multi-stem pre-miRNAs, respectively [24]. However, because there were only 19 (~5.5%) known multi-stem pre-miRNAs in zebrafish, we simply mixed single- and multi-stem pre-miRNAs together for the feature selection. First, we collected 195 sequence and structure features from previously published studies [21,24,46]. We also introduced 11 new structure features, including the ratio of paired nucleotides, the ratio of unpaired nucleotides, the number of bulges, the normalized number of bulges, maximum number of consecutive paired nucleotides, average unpaired nucleotides per bulge, MFE4, MFE5, the size of largest bulge, the normalized number of stems, and the normalized number of loops. Because exhaustively searching optimal combination of 206 features is too time-consuming, we classified these features into 23 sets. The first set contains 16 sequence features of dinucleotide frequencies from microPred [46], and the second set includes 32 structure features of triplet elements raised by triplet-SVM [21]. The third set contains 138 structure features for specifically predicting multi-stem pre-miRNAs in miRD [24]. The remaining unclassified 20 sequence or structure features were directly regarded as 20 sets.

Then F-score [29], a simple measurement for the feature selection, was used to rank the feature sets. The F-score of the *i*th feature set was defined as:

$$F\left(i\right)\equiv \frac{{\left({\bar{x}}_{i}^{\left(+\right)}-{\bar{x}}_{i}\right)}^{2}+{\left({\bar{x}}_{i}^{\left(-\right)}-{\bar{x}}_{i}\right)}^{2}}{\frac{1}{n_{+}-1}\sum_{k=1}^{n_{+}}{\left(x_{k,i}^{\left(+\right)}-{\bar{x}}_{i}^{\left(+\right)}\right)}^{2}+\frac{1}{n_{-}-1}\sum_{k=1}^{n_{-}}{\left(x_{k,i}^{\left(-\right)}-{\bar{x}}_{i}^{\left(-\right)}\right)}^{2}},$$

where *n*_*+*_ and *n*_*−*_ are the numbers of positive and negative samples, respectively; ${\bar{x}}_{i}$, ${\bar{x}}_{i}^{\left(+\right)}$ and ${\bar{x}}_{i}^{\left(-\right)}$ are the average of the *i*th feature set of total, positive and negative samples, respectively. $x_{k,i}^{\left(+\right)}$ and $x_{k,i}^{\left(-\right)}$ are the *i*th feature set of the *k*th positive sample and negative sample, respectively. For 20 sets containing only one feature, the F-scores were directly computed. For three sets with multiple features, the F-score value of each feature was calculated separately, and then the average F-score of each set was calculated, respectively. Finally, 19 feature sets including 17 sequence and 48 structure features were selected to build an SVM model for pre-miRNA prediction.

As an efficient machine learning algorithm, SVMs map original data to a high-dimensional feature space, and seek an optimal hyperplane to separate the positive and negative samples. The decision function of SVMs is

$$y=\mathrm{sgn}\left(\sum_{i=1}^{N}\gamma_{i}\alpha_{i}\kappa \left(x,x_{i}\right)+b\right),$$

where *κ* is a kernel function, and parameters *α*_*i*_ are the coefficients to be learned through maximizing

$$\begin{array}{l} \sum_{i=1}^{N}\alpha_{i}-\frac{1}{2}\sum_{i,j=1}^{N}\alpha_{i}\alpha_{j}\gamma_{i}\gamma_{j}\kappa \left(x_{i},x_{j}\right),\mathrm{subject}\;\mathrm{to}\;\\ \sum_{i=1}^{N}\alpha_{i}\gamma_{i}=0\;\mathrm{and}\;0\leq \alpha_{i}\leq c\;\left(i=1,\ldots ,N\right) \end{array}$$

The LIBSVM package was used for training (version 3.16, http://www.csie.ntu.edu.tw/~cjlin/libsvm/), whereas the svm-scale program in LIBSVM was used to rescale all features with the interval [−1.0, 1.0]. The most widely used radial basis function (RBF) was chosen. The penalty parameter *C* and RBF kernel parameter γ were exhaustively searched as 8.0 and 0.03125, respectively.

### Performance evaluation

Among the predicted positive results obtained by ZmirP, the real positives are called true positives (*TP*), while others are called false positives (*FP*). Among the predicted negative results obtained by ZmirP, real negatives are called true negatives (*TN*), while the others are called false negatives (*FN*). In a classification problem, the specificity (*Sp*), sensitivity (*Sn*), accuracy (*Ac*) and Mathew’s correlation coefficient (*MCC*) are most widely used to evaluate the prediction system. They are defined as:

$$\begin{array}{l} \mathrm{Sn}=\frac{\mathrm{TP}}{\mathrm{TP}+\mathrm{FN}},\;\mathrm{Sp}=\frac{\mathrm{TN}}{\mathrm{TN}+\mathrm{FP}},\\ \mathrm{Ac}=\frac{\mathrm{TP}+\mathrm{TN}}{\mathrm{TP}+\mathrm{FP}+\mathrm{TN}+\mathrm{FN}} \end{array}$$

and

$$\mathrm{MCC}=\frac{\left(\mathrm{TP}\times \mathrm{TN}\right)-\left(\mathrm{FN}\times \mathrm{FP}\right)}{\sqrt{\left(\mathrm{TP}+\mathrm{FN}\right)\times \left(\mathrm{TN}+\mathrm{FP}\right)\times \left(\mathrm{TP}+\mathrm{FP}\right)\times \left(\mathrm{TN}+\mathrm{FN}\right)}}$$

In this study, the LOO validation and 4-, 6-, 8-, 10-fold cross-validations were performed as previously described [47]. And the Receiver Operating Characteristic (ROC) curves were plotted.

### Small RNA library construction and sequencing

Breeding wild-type zebrafish (*Danio rerio*) (AB type) were maintained under standard library conditions and the embryonic stages in this study were as described [15,48]. Zebrafish embryos were collected at 1-cell (0.2 hpf), 16-cell (1.5 hpf), 512-cell (2.75 hpf), oblong (3.7 hpf), 5.3 hpf (50% epibody), 6-somite (12 hpf), 24 hpf (day1) and 48 hpf (day2) stages. Total RNA from embryos was isolated using Trizol reagent (Invitrogen). RNAs were fractioned on 15% denaturing polyacrylamide gels, and small RNAs were isolated and purified. Subsequently, small RNAs were ligated with both a 5′ adapter and 3′ adapter for reverse transcription using SuperscriptTM II reverse transcription kit (Invitrogen) following the manufacturer’s instructions at 42°C for 1 h and 70°C for 15 min. After that, the reverse transcribed product, cDNA was amplified by the following PCR program: a 15-cycle reaction at 98°C for 30 sec, followed by 15 cycles consisting of 10 sec at 98°C, 15 sec at 72°C, and then 10 min at 72°C. After obtaining a 92 bp DNA band on 6% denaturing PAGE gels, the PCR products were enriched by ethanol precipitation and purified using Spin-X filter columns (Fisher). Finally, small RNA libraries were constructed and sequenced by the Illumina HiSeq™ 2000 following the manufacturer’s protocol in BGI-Shenzhen, China. The primers

3′ ligation adaptor: 5′-GUUCAGAGUUCUACAGUCCGACGAUC-3′

5′ ligation adaptor: 5′-PUCGUAUGCCGUCUUCUGCUUGUidT-3′

PCR forward primer: 5′-CAAGCAGAAGACGGCATACGA-3′

PCR reverse primer:

5′-AATGATACGGCGACCACCGACAGGTTCAGAGTTCTACAGTCCGA-3′

Raw reads obtained from sequencing platform were processed by filtering out the low quality reads, trimming 3′ adaptor sequences, removing the 5′ adaptor contaminants, and eliminating reads containing ploy(A).

### Quantitative real-time PCR

Quantitative real-time PCR (qRT-PCR) assays (miScript Reverse Transcription Kit and miScript SYBR Green PCR Kit, Qiagen) were performed to determine the expression levels of four known miRNAs (dre-miR-456, dre-miR-22a, dre-miR-206 and dre-miR-192). 10 ng of total RNA extracted from zebrafish embryos was used for cDNA synthesis. U6 snRNA was used as an endogenous control and each reverse transcription was conducted in triplicate. The expression levels of miRNAs were measured by the threshold cycle values (C_t_). The relative expression levels were assigned as Equation 2^-∆∆ct^. The primers were designed as follows (Universal reverse primer was brought in miScript SYBR Green PCR Kit of Qiagen):

dre-miR-456_F: 5′-CAGGCTGGTTAGATGGTTGTCA-3′

dre-miR-22a_F: 5′-GCTGCCAGCTGAAGAACTGTAAA-3′

dre-miR-206_F: 5′-TGGAATGTAAGGAAGTGTGTGGA-3′

dre-miR-192_F: 5′-GTGATGACCTATGAATTGACAGCC-3′

U6 snRNA_F: 5′-CTCGCTTCGGCAGCACA-3′

### Northern blots

A total of 200 μg of total RNA was extracted from zebrafishes embroys at 16-cell stage, electrophoresed on 1.2% denaturing agarose-formaldehyde gel, and transferred to HybondTM-N + nylon membranes (Amersham, USA) for Northern blot analysis of three novel miRNAs, m0027-5p, chr6_7844-5p and m0026-5p. U6 snRNA was used as an endogenous control. The sequences of northern probes were 3′-DIG-labelled and complementary to the novel mature miRNAs and U6 sequences. The probes were as follows:

m0027-5p: 5′-CACGTCCCCCCAGAATTCACCATAAC-3′ DIG

chr6_7844-5p: 5′-CACCCTCATCTCCCCAAACCTACAC-3′ DIG

m0026-5p: 5′-AAGTGAGTCTCGTCATTCTGTC-3′ DIG

U6 snRNA: 5′-CTCGCTTCGGCAGCACA-3′ DIG

All of the studies using zebrafish were approved by the Animal Care and Use Committee of Huazhong University of Science and Technology.

### Accession numbers

The sequencing data reported in this work can be available from the NCBI Short Read Archive (SRA) under accession number [SRP028862].

## Abbreviations

sRNAs: small non-coding RNAs; miRNAs: MicroRNAs; piRNAs: Piwi-interacting RNAs; pri-miRNAs: primary miRNA molecules; dsRNAs: double-stranded RNAs; RISC: RNA-induced silencing complex; hpf: hours post fertilization; MZT: Maternal-to-zygotic transition; NGS: Next-generation sequencing; dpf: days post fertilization; triplet elements: local structure-sequence features; SVMs: Support vector machines; RF: Random forest; MFE: Minimum of free energy; kNN: *k*-nearest neighbors; ZmirP: Zebrafish miRNA prediction; CSZ: Characterization of small RNAome for zebrafish; RBF: Radial basis function; TP: True positive; FP: False positive; TN: True negative; Sp: Specificity; Sn: Sensitivity; Ac: Accuracy; MCC: Mathew’s correlation coefficient; ROC: Receiver Operating Characteristic; qRT-PCR: quantitative real-time PCR; RPM: reads per million; Non-repeat-associated piRNAs: piRNAs that were not annotated as repeat sequences; Repeat-associated piRNAs: piRNAs that could be mapped to repetitive sequences.

## Competing interests

The authors declare that they have no competing interests.

## Authors’ contributions

YX and HJ designed the project; YY, YX developed the algorithm and analyzed data; QY, LM and QJ performed experiments; WD, ZL, YZ and JR extensively shared and discussed data; YX, YY and QY wrote the manuscript. All authors read and approved the final manuscript.

## Supplementary Material

Additional file 1: Table S1 The detailed data statistics for different type of reads in eight libraries. Click here for file

Additional file 2: Table S2 The 218 known zebrafish miRNAs identified from the sRNA-seq data, with corresponding mappable reads. Click here for file

Additional file 3: Table S3 The 25 predicted miRNAs. Click here for file

Additional file 4: Figure S1 The secondary structures of 25 potentially novel miRNAs. Click here for file

Additional file 5: Table S4 The piRNA clusters identified from the sRNA-seq data. Click here for file

Additional file 6: Figure S2 The distribution of different types of sRNAs in data from Wei’s data [19]. (A) The proportion of total mappable reads for different types of sRNAs; (B) The distribution of unique mapped reads for different types of sRNAs. Click here for file
