# Supplementary figures and images for: Systematic characterization of small RNAome during zebrafish early developmental stages
Source: BMC Genomics. 2014 Feb 10;15:117. doi: 10.1186/1471-2164-15-117 (PMC3932949; doi:10.1186/1471-2164-15-117)

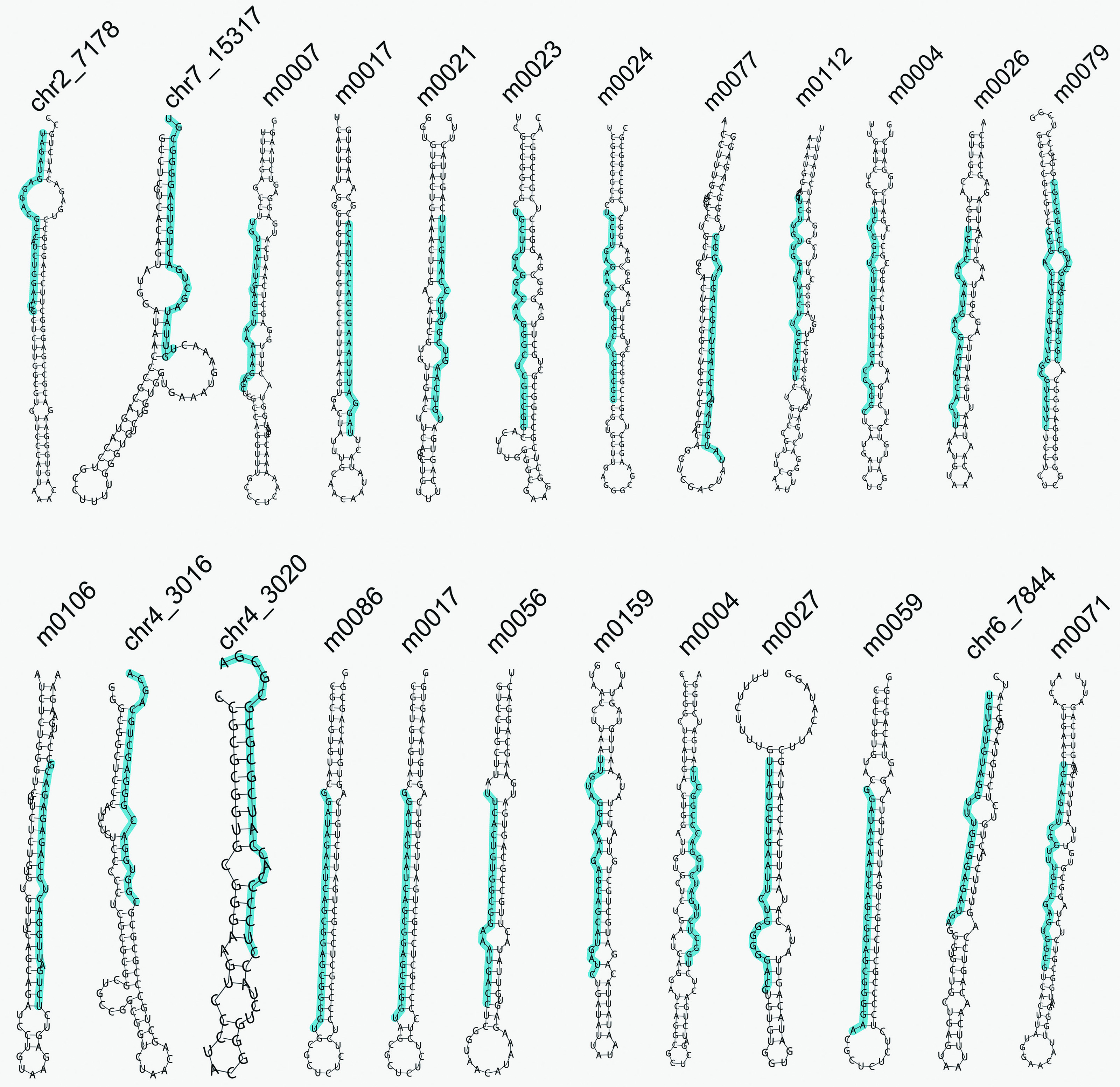

Supplement: Additional file 4: Figure S1 — The secondary structures of 25 potentially novel miRNAs. [file 1471-2164-15-117-S4.jpeg]

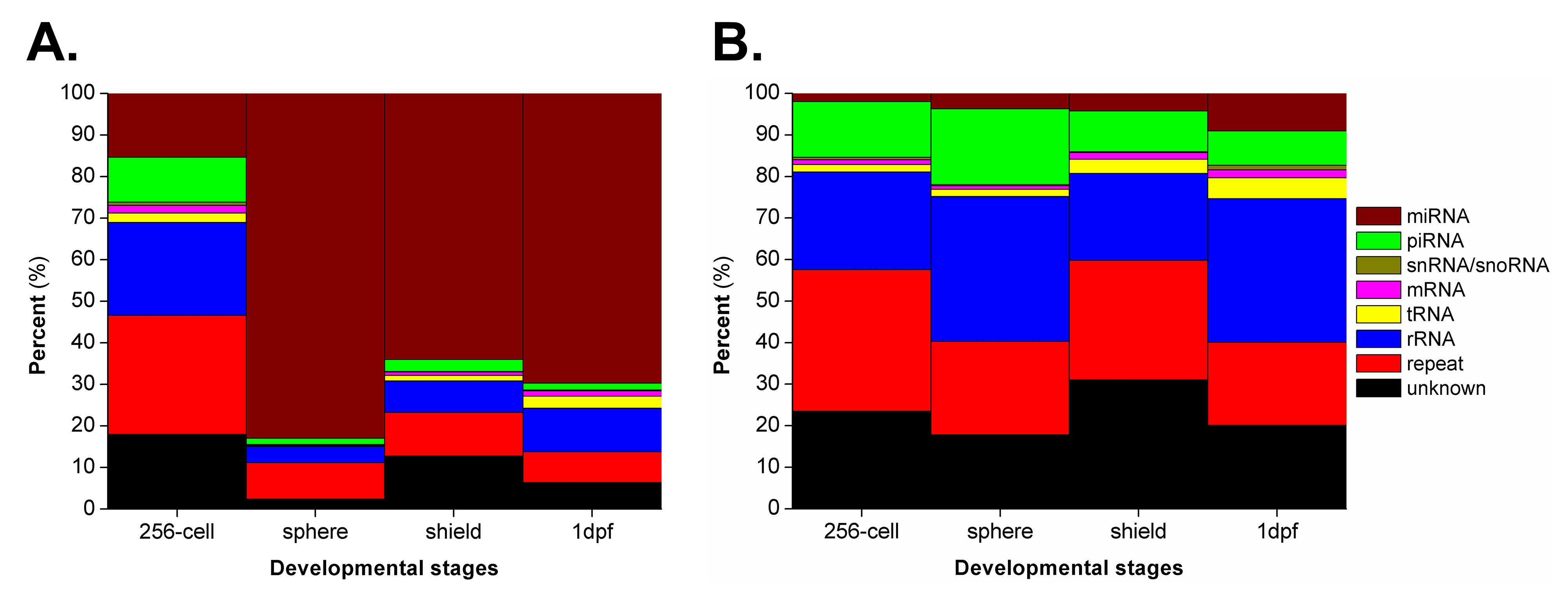

Supplement: Additional file 6: Figure S2 — The distribution of different types of sRNAs in data from Wei’s data [19]. (A) The proportion of total mappable reads for different types of sRNAs; (B) The distribution of unique mapped reads for different types of sRNAs. [file 1471-2164-15-117-S6.jpeg]
